# BK channel overexpression on plasma membrane of fibroblasts from Hutchinson-Gilford progeria syndrome

**DOI:** 10.18632/aging.101621

**Published:** 2018-11-06

**Authors:** Isabella Zironi, Entelë Gavoçi, Giovanna Lattanzi, Angela Virelli, Fabrizio Amorini, Daniel Remondini, Gastone Castellani

**Affiliations:** 1 Department of Physics and Astronomy (D.I.F.A.), University of Bologna, Bologna , Italy; 2 Interdepartmental Centre “L. Galvani” for integrated studies of Bioinformatics, Biophysics and Biocomplexity (C.I.G.), University of Bologna, Bologna, Italy; 3 CNR Institute of Molecular Genetics, Unit of Bologna, Bologna, Italy; 4 Rizzoli Orthopedic Institute, Bologna, Italy; 5 National Institute for Nuclear Physics (INFN), Bologna, Italy

**Keywords:** Hutchinson-Gilford Progeria Syndrome (HGPS), *LMNA* gene, aging, patch clamp, membrane channels, K^+^ current, cellular proliferation

## Abstract

Hutchinson-Gilford progeria syndrome (HGPS) is an extremely rare genetic disorder wherein symptoms resembling aspects of aging are manifested at a very early age. It is a genetic condition that occurs due to a *de novo* mutation in the *LMNA* gene encoding for the nuclear structural protein lamin A. The lamin family of proteins are thought to be involved in nuclear stability, chromatin structure and gene expression and this leads to heavy effects on the regulation and functionality of the cell machinery. The functional role of the large-conductance calcium-activated potassium channels (BK_Ca_) is still unclear, but has been recently described a strong relationship with their membrane expression, progerin nuclear levels and the ageing process. In this study, we found that: i) the outward potassium membrane current amplitude and the fluorescence intensity of the BK_Ca_ channel probe showed higher values in human dermal fibroblast obtained from patients affected by HGPS if compared to that from healthy young subjects; ii) this result appears to correlate with a basic cellular activity such as the replicative boost. We suggest that studying the HGPS also from the electrophysiological point of view might reveal new clues about the normal process of aging.

## Introduction

Hutchinson–Gilford progeria syndrome (HGPS) is a rare fatal genetic disorder characterized by various clinical features and phenotypes of premature ageing. The disorder has a very low incidence rate, occurring in an estimated 1 per 8 million live births. Children born with HGPS typically appear normal at birth, but within a year, they begin to display symptoms related to a global premature aging phenotype including hair loss, diminished subcutaneous fat, cardiovascular diseases and skeletal abnormalities. The notable thing in HGPS is that there is no apparent alteration of the central nervous system. On average, death occurs at the age of 13 from stroke, myocardial infarction, heart failure or atherosclerosis [1,2]. HGPS is caused by accumulation of an alternative splice variant of lamin A, progerin, which results from erroneous activation of a cryptic splice site within the *LMNA* gene [3–5]. Progerin is a truncated form of prelamin A, the lamin A precursor, which is also accumulated, as full-length protein, in HGPS and other laminopathies and affects nuclear organization, chromatin dynamics, regulation of gene expression and epigenetic regulation [6–10]. Previous studies conducted in primary dermal fibroblasts obtained from HGPS patients, describe a progressive impairment of cell proliferation and cell cycle exit with increasing expression of progerin [11–14] pointing the truncated form of prelamin A as responsible for the accelerated senescence phenotype [15]. Progerin has been also detected in normal ageing cells [11,12,16] where its accumulation leads to acceleration of senescence [15]. However, the detailed cellular mechanisms causing clinical phenotype of HGPS is still unclear.

Using a systems-biology approach Sokolowski and colleagues demonstrated that large-conductance calcium-activated potassium (BK_Ca_) expression decreased with the silencing of the *LMNA* gene [17]. The existence of putative binding sites for life/death signals that could alter BK_Ca_ expression indicates an interactive role with *LMNA* on cell viability. We recently observed that total K^+^ current amplitude recorded from normal hDF membrane decreases with ageing, while it is partially recovered in cells obtained from centenarian subjects. This effect was mainly mediated by the relative remodeling of BK_Ca_ and Kv1.1 channel expression on plasma membrane [18], suggesting for these channels a possible role as potential markers for early diagnosis of ageing-related pathologies. BK_Ca_ channels are found in almost all excitable and non-excitable cells and are involved in a broad number of physiological processes, mostly cell type- and tissue-dependent including action potential repolarization, resting membrane potential, cytosolic calcium (Ca^2+^) concentrations and cell volume regulation, hormone secretion and blood pressure control [19,20]. In particular, in human cardiac fibroblasts they are responsible for the modulation of the rhythmic oscillation of the membrane potential promoting proliferation throughout cell cycle [21]. There are several evidences that variations in the activity or expression of BK channels are linked to pathophysiological disorders related to aging, such as cardiovascular disorders, cancer, diabetes and neurological diseases [22]. In particular, it has been shown that the coronary arteries of elderly human subjects exhibit a profound loss of BKα and an inability to oppose vasoconstriction [23], which paved the way to clinical trials using pharmacological activators designed to increase the open-state probability of the channel [24].

In the present work we aimed to investigate how specific cellular features interacting to each other such as membrane channel activity, calcium concentration and proliferation boost are affected by *LMNA*. Furthermore, by a closer view of these parameters, we might add knowledge on HGPS cell behavior and provide further evidences that recapitulate physiological mechanisms of ageing [12,25].

## RESULTS

### Potassium outward current through BKCa channels drastically increases in HGPS affected cells

**Figure 1:**
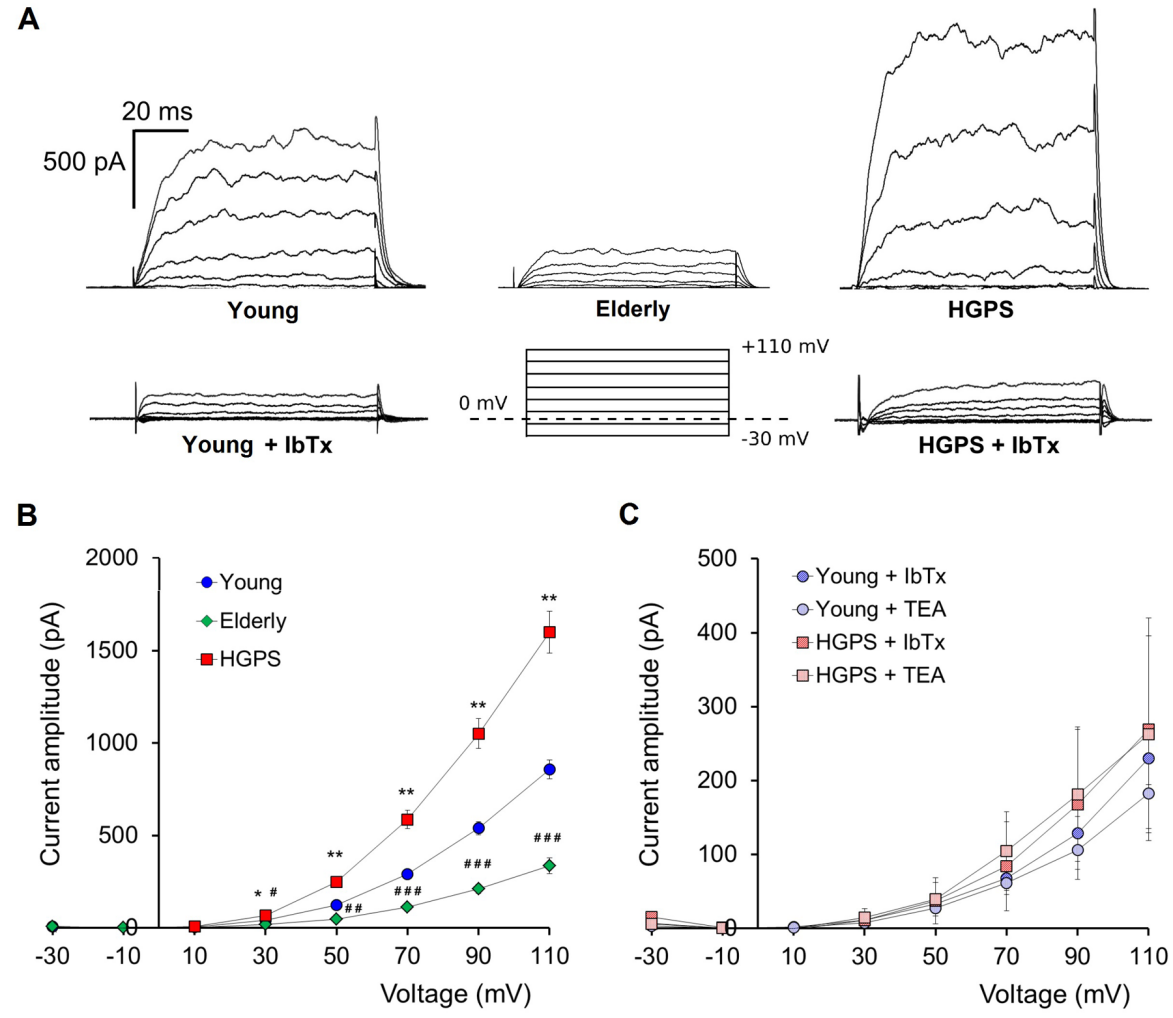
**Outward currents patch-clamp recorded in whole-cell configuration.** (**A**) Representative examples of current traces recorded in hDF obtained from a young donor, an elderly, and a patient affected by HGPS. Current traces recorded after 100 nM IbTx application and a graphical representation of the pulse protocol (holding potential at 0 mV) are also shown. (**B**) Average ± SEM of current-voltage relationships (I–V) recorded in hDF obtained from healthy donors (Young, n=83; Elderly, n=16) and patients affected by HGPS (n=80). (**C**) Average ± SEM of current-voltage relationships (I–V) recorded in hDF obtained from young donors and patients affected by HGPS treated by 100 nM IbTx (n=6) and 10 mM TEA (n=4). Young vs. HGPS: *p<0.05; **p<0.01; Young vs. Elderly: #p<0.05; ##p<0.001; ###p<0.0001.

We compared the whole-cell outward currents of primary hDF cells obtained from the HGPS group with those obtained from the two control groups: Young and Elderly. In all groups of cells the recorded currents had the properties described in Zironi et al. [18], that is: noisy traces due to the large conductance of the BK_Ca_ channels, activation at about 10 mV and a semi-complete inhibition after the loading with the specific blocker Iberiotoxin (IbTx 100 nM, a concentration known to completely inhibit BK_Ca_ channels activity) (Fig. 1A-B). To determine the weight of the BK_Ca_ component on the whole-cell outward currents we also recorded after perfusion with a solution containing the non-selective K^+^ channels blocker Tetraethylammonium (TEA 10 mM, Fig. 1C). The results confirmed that, in all the groups considered, the outward currents are mostly gated through the BK_Ca_ channels (Fig. 1).

The current amplitude analysis conducted on the traces obtained with the step protocol (see Material and Methods) revealed that, starting from 30 mV of stimulus, in the Elderly group the whole-cell outward current is significantly lower compared to Young, while, surprisingly, the HGPS group showed the highest values (Fig. 1A-B). We also observed that HGPS group’s data are characterized by a very high standard deviation suggesting that there is a strong difference in terms of amount of current within this population. The analysis conducted as described in Materials and Methods and summarized in Table 1 indicated that the majority of cells recorded in the HGPS group shows a current amplitude higher than the average from the Young group. For each cell, we evaluated the current density by normalizing the current amplitude to the membrane capacitance. The results (reported in Table 2) excluded that the increase of BK_Ca_ channels weight on eliciting the outward current fluxes might be affected by the cell size.

**Table 1:** Percentage of cells from the HGPS group expressing a mean current amplitude defined as in Materials and Methods.

**% of HGPS hDF** (n=80)		
**Low**	**Intermediate**	**High**
6.25	38.75	55.00

**Table 2:** Summary of current amplitudes, capacitances, radius and current density obtained from each group at 110 mV of stimulation step.

	**Young** n=83	**Elderly** n=16	**HGPS** n=80
**Mean Amplitude** (pA) ± SEM	857.6 ± 50.7	336.9 ± 41.6	1599.9 ± 112.9
**Young**		p<0.0001	p<0.05
**Elderly**	p<0.0001		p<0.0001
**HGPS**	p<0.05	p<0.0001	
**Capacitance** (pF)	41.0 ± 2.0	30.1 ± 2.2	36.2 ± 1.3
**Radius** (μm)	18.1 ± 0.4	15.2 ± 0.4	17.0 ± 0.3
**Mean Current density** (pΑ/pF) ± SEM	28.6 ± 3.0	12.1 ± 1.7	53.2 ± 5.1
**Young**		p<0.01	p<0.05
**Elderly**	p<0.01		p<0.01
**HGPS**	p<0.05	p<0.01	

p values calculated by the Student’s *t*-test and corrected by the Bonferroni test for multiple comparison.

### Expression of BK_Ca_ channels on plasma membrane increases in HGPS while decreases with ageing

**Figure 2:**
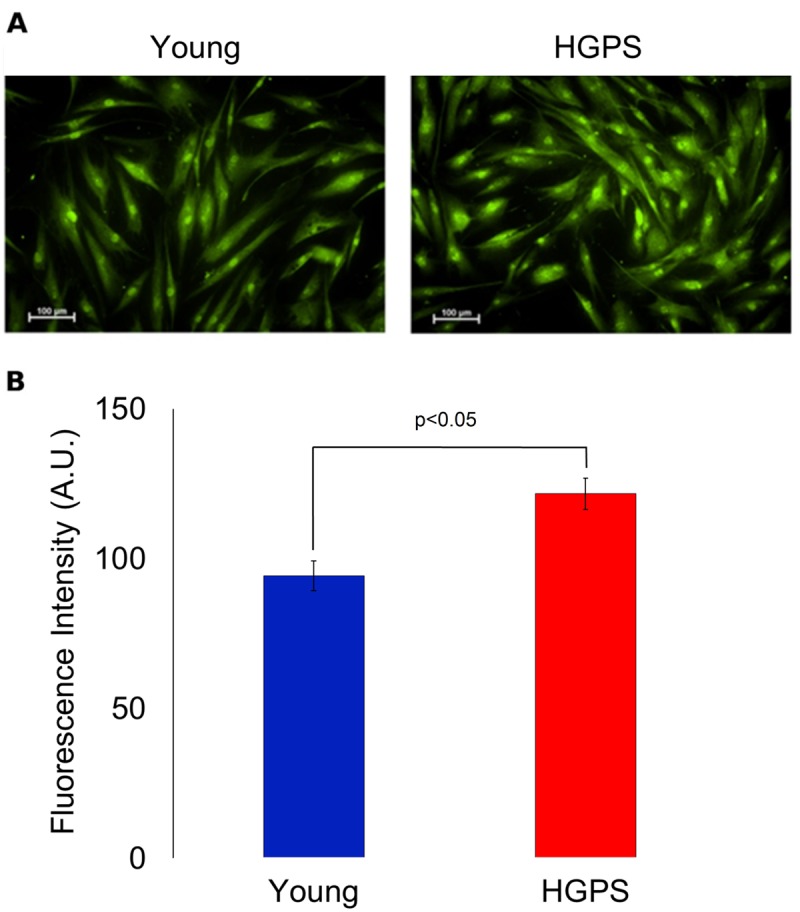
**Immunofluorescence detection for BK_Ca_ channels expressed on plasma membrane in fixed cells.** (**A**) Fluorescence micrographs of isolated hDF obtained from young and HGPS donors incubated with an anti-BK_Ca_ α subunit primary antibody visualized by FITC-conjugated secondary antibody and acquired at 200× magnification. Scale bars: 100 μm. (**B**). Quantification of mean fluorescence intensity of anti-BK_Ca_ antibody-stained cells. The green fluorescence intensity values are obtained from 30 cells (A.U. ± SEM). Significant differences calculated according to the Student’s t-test (p<0.05) are indicated.

**Figure 3:**
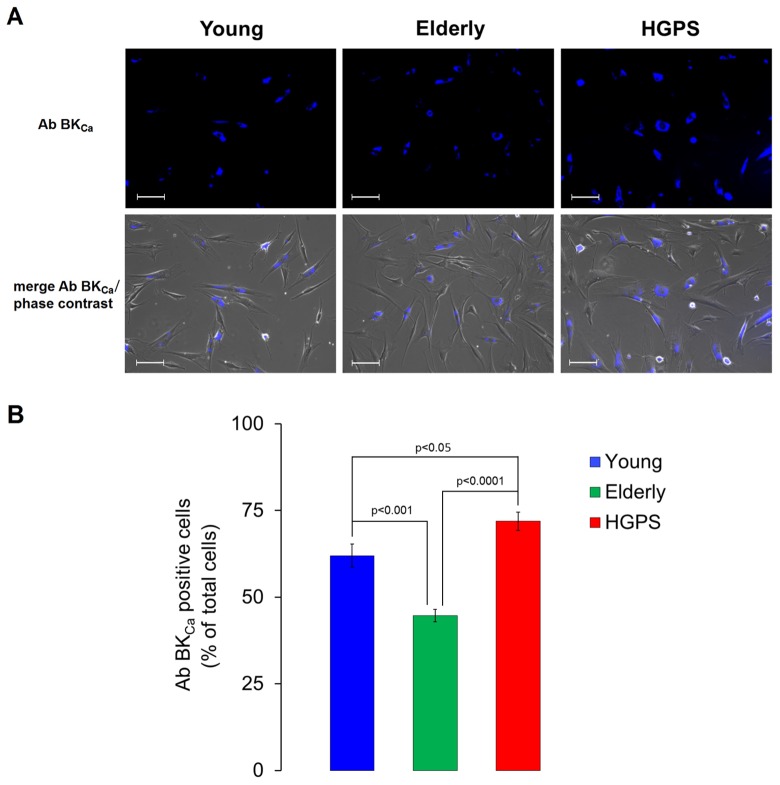
**Immunofluorescence detection for BK_Ca_ channels expressed on plasma membrane in living cells.** (**A**) Fluorescence and fluorescence/phase contrast merged micrographs of isolated hDF obtained from young, elderly and HGPS donors incubated with an anti-BK_Ca_ α subunit primary antibody visualized by the conjugated Alexa Fluor 350 fluorophore and acquired at 200× magnification. Scale bars: 100 μm. (**B**) Histogram showing the percentage of cells expressing a blue fluorescence intensity over a fixed threshold (% ± SEM). Significant differences calculated according to the Student’s t-test (p<0.05) are indicated.

**Figure 4:**
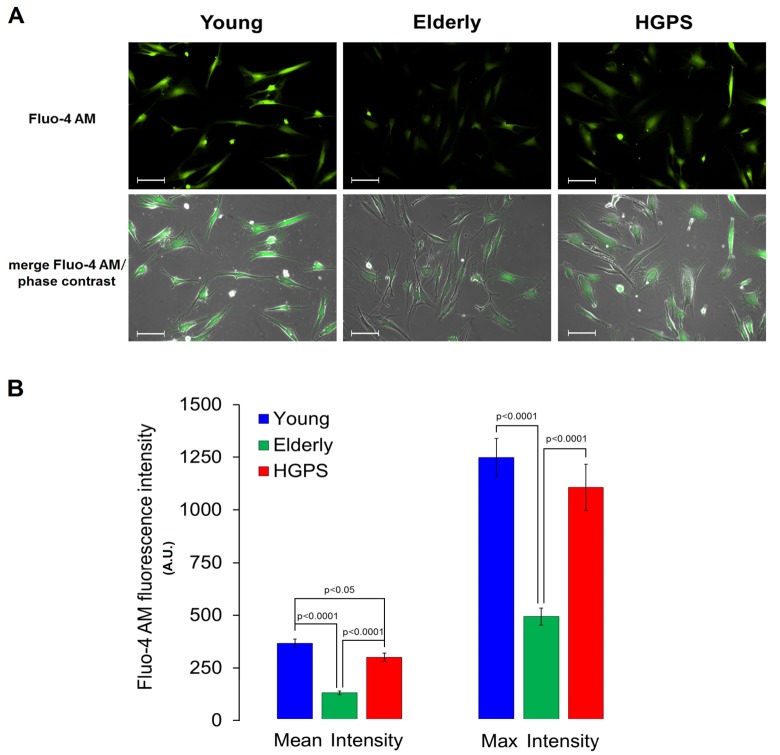
**Detection of calcium concentration by fluorescence in living cells**. (**A**) Fluorescence and fluorescence/phase contrast merged micrographs of isolated hDF obtained from young, elderly and HGPS donors incubated with the cell-permeant Ca^2+^ indicator Fluo-4 AM (2 μM) and at 200× magnification. Scale bars: 100 μm. (**B**) Quantification of Mean and Max fluorescence intensity (A.U. ± SEM). Significant differences calculated according to the Student’s t-test (p<0.05) are indicated.

A semi-quantitative analysis of the large conductance Ca^2+^-activated K^+^ channel was assessed using a polyclonal IgG against an extracellular peptide of the α-subunits in hDF obtained from the Young and HGPS groups. The results showed in Figure 2 indicate that HGPS hDF express more BK_Ca_ channels compared to Young, being the mean fluorescence intensity of the HGPS group about 30% higher. In this case, the samples were fixed by methanol as described in Materials and Methods, but in order to confirm these results in physiological conditions, an immunofluorescence assay in living cells was performed. Data shown in Figure 3 indicate that the percentage of cells expressing BK_Ca_ channels on the membrane above a fixed threshold is significantly higher in the HGPS group as compared to the two control groups (Young and Elderly). Since the elicited current amplitudes are recorded while BK_Ca_ channels are totally open (membrane are clamped at an over-saturating voltage potential of +110 mV) knowing the cellular membrane potential (V_mem_), the Ohm’s law can be solved to calculate the total K^+^ conductance which depends on both the number and yield of the channels. Furthermore, considering that BK_Ca_ channel activity is also calcium-dependent, a Ca^2+^ assay detection has been done. As shown in Figure 4 the mean and the maximum fluorescence intensity of the Fluo-4AM probe are both significantly lower in the Elderly group when compared to the Young and HGPS groups (p<0.0001), while is significant only the mean intensity when the HGPS group is compared to the Young one (p<0.05). This observation indicates that the intracellular Ca^2+^ concentration decrease with normal aging while in the pathophysiological disorder related to aging HGPS it does so only in some cells.

Based on the whole evaluation of electrophysiology and immunofluorescence results, it can be claimed that the increase of the whole-cell outward current for the HGPS group compared to the Young can be mainly ascribed to a BK_Ca_ channels overexpression on plasma membrane rather than an increment of their activity Ca^2+^ -modulated. Interestingly, this phenomenon does not occurs during the physiological process of aging, since hDF obtained from elderly subjects are conversely characterized by a decrease in BK_Ca_ channels expression on plasma membrane and intracellular Ca^2+^ concentration.

### Proliferation capability impairment in HGPS hDF involve BK_ca_ channels

**Figure 5:**
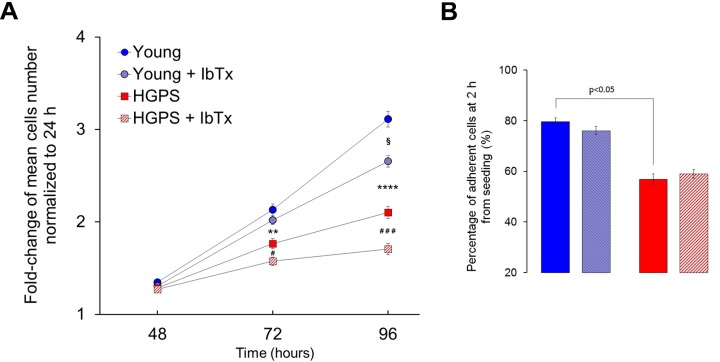
**Proliferation and adhesion rates without and with the BK_Ca_ inhibitor IbTx.** (**A**) Average number of hDF obtained from young healthy donors and patients affected by HGPS estimated at 48, 72 and 96 hours from seeding and normalized at 24 h (fold-change ± SEM). Healthy and HGPS hDF were allowed to proliferate untreated (Young n=54; HGPS n=36) and treated by 100 nM IbTx (Young n=53; HGPS n=36). Young vs. HGPS: **p<0.001; ****p<5×10^-7^; Young vs. Young + IbTx: §p<0.01; HGPS vs. HGPS + IbTx: #p<0.01; ###p<0.0001. (**B**) Average percentage ± SEM of adherent hDF cells counted 2 h after seeding, treated (Young n=35, HGPS n=22) and untreated by 100 nM IbTx (Young n=40, HGPS n=24); image legend is the same as Figure A.

The proliferation rate of hDF obtained from the Young and HGPS groups has been assessed by counting the number of cells at 24, 48, 72 and 96 hours from seeding. The fold-change of the mean number of cells normalized to the first 24 h is reported in Figure 5A. The statistical evaluation of results indicates that replication rate is significantly reduced in HGPS cells compared to Young at 72 h (p<0.001) and at 96 h (p<5×10^-7^). Since the adhesion process is a necessary step towards proliferation (a lower capability to adhere cause a reduction in the number of the initially seeded population), the same population of cells from the Young and HGPS groups was monitored two hours after seeding. The analysis revealed that the percentage of adherent HGPS hDF is about 20% lower (p <0.05) compared to that obtained from the Young group (Fig. 5B). To account whether BK_Ca_ channels are involved in the process, we performed cell growth and adhesion experiments keeping cells in replicative conditions from 0 to 96 hours in the presence or not (treated or untreated cells) of 100 nM IbTx. The statistical analysis showed that the proliferation rate of Young and HGPS cells in comparison to that of same-group untreated cells at 72 h from seeding is significantly lower only for the HGPS group (p<0.01), while at 96 h it is for both groups with p values of <0.01 for the Young group and <0.0001 for the HGPS one (Fig. 5A). Conversely, the adhesion capability of HGPS cells is not significantly affected by the BK_Ca_ channels block (Fig. 5B). Therefore, the inhibition of BK_Ca_ channels activity by the specific toxin IbTx negatively affects only the proliferation rate and the effect seems to be more consistent in HGPS cells compare to those Young.

These results indicate that the proliferation is impaired in HGPS hDF and necessarily involves the BK_Ca_ channels activity, while the detrimental effect on adhesion may depend on other causes associated with the pathological state of the HGPS cells. Moreover, comparing these results with data on current amplitudes (Fig. 1) it can be argued that a lower current amplitude corresponds to a higher proliferation rate and vice-versa by suggesting a causal relationship between the electrophysiological properties of the cells and their proliferation boost.

### Senescence rate in hDF from elderly group is the highest between groups

**Figure 6:**
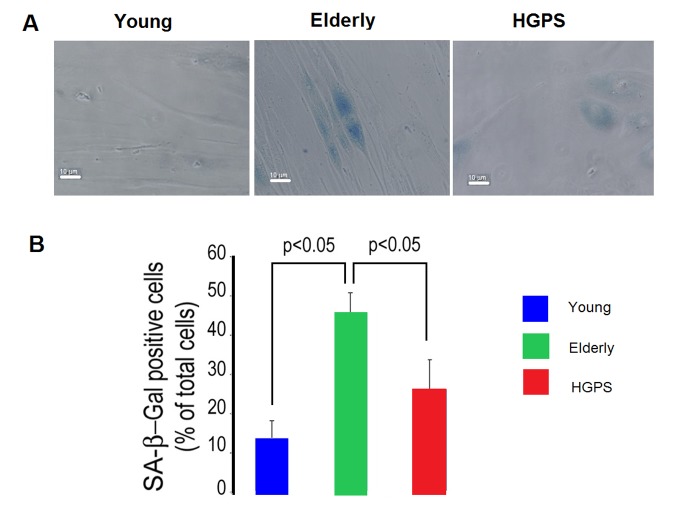
**Percentage of senescent cells.** (**A**) Representative micrographs of isolated hDF obtained from young, elderly and HGPS donors. Cells with blue staining indicated positive for SA β-galactosidase activity. Images acquired in transmission light bright field at 400× magnification. Scale bar: 10 μm. (**B**) The percentages of positive hDF from Young, Elderly and HGPS groups are reported in the graph as mean value of three independent staining (% ± STD). Significant differences calculated according to the Student’s t-test (p<0.05) are indicated.

As shown in Figure 6, relative to the Young group the percentage of hDF positive to the senescence-associated β-galactosidase (SA β−gal) staining is higher in cells from the Elderly group, while a tendency to increase is observed in HGPS cells, yet not statistically significant. Interestingly, the percentage of senescent cells in the HGPS group is significantly lower than that observed in the Elderly one.

## DISCUSSION

The evidences described in this study can be summarized as follows: a) human dermal fibroblasts (hDF) obtained from patients carrying a mutation of the *LMNA* gene (c. 1824C>T, p.G608G) overexpress BK_Ca_ channels on plasma membrane and display an impaired proliferation rate; b) in vivo aged cells, obtained from healthy elderly subjects are characterized by very low K^+^ currents and high percentage of senescent cells.

The BK_Ca_ channel expressed on hDF membrane consists of pore-forming, voltage- and calcium-sensing α subunits in association with regulatory subunits. The α-subunits are encoded by a single gene (*KCNMA1*), which undergoes extensive alternative pre-mRNA splicing leading to a different complement of BK_Ca_ channel α-subunits and providing tissue-specific features of activity, trafficking and regulation [26]. As other systemic laminopathies, HGPS is characterized by impressive alterations in the nuclear profile and in chromatin organization, due to accumulation of unprocessed prelamin-A. Because the lamin A serves as a docking site for transcription factors and chromatin-associated proteins, a dysfunctional lamin, like progerin, may influence gene expression in several ways [17,27,28]. This might in turn affect the encoding process of BK_Ca_ channels in one or more steps, even indirectly, acting for example on support proteins that participate in the assembly of the channel on plasma membrane, leading in general to a non-programmed modulation of the BK_Ca_ channel protein expression.

BK_Ca_ channel is the main type expressed on the hDF membrane. Thanks to its large conductance for out-ward K^+^ fluxes and to its sensitivity for voltage variation and Ca^2+^ concentration it takes a gating role on setting the V_mem_. Moreover, we know that changes of the membrane potential play a crucial role in several cellular pathways [29], including cell cycle and proliferation. For example, in hDF, but also in other cell types (lymphocytes, astrocytes, and Schwann cells), it has been proven that the modulation of V_mem_ is required for both G1/S and G2/M phase transitions. Hyperpolarization serves for G1/S progression while depolarization is necessary for the G2/M transition. This defines a model that outlines a rhythmic oscillation of membrane potential throughout the cell cycle, with a spike in hyperpolarization, occurring before DNA synthesis, followed by a prolonged period of depolarization necessary for mitosis [20]. Voltage dependent K^+^ channels, including BK_Ca_ channels are the main responsible of this modulation [30]. In fact, it has been shown that their expression and activity change across cell cycle stages [19,31] indicating a key regulatory role in the proliferation process.

Within this perspective, is very important to relate the BK_Ca_ channels functionality of HGPS cells to their V_mem_ and then to their proliferation functionality. The overexpression of the BK_Ca_ channels α subunit observed in HGPS cells results in a greater outflow of K^+^ in response to physiological depolarization of the cell membrane. This may elicit a feedback effect that tends to set a more polarized electric state with respect to the healthy cells, and therefore making further depolarization, the condition required for mitosis, more difficult, thus resulting in a decrease of the proliferation rate. By testing the BK_Ca_ channels activity on the proliferation process with the selective inhibitor IbTx we observed a further decrease in the replicative boost of HGPS hDF. Probably, in this case, the out-flowing K^+^ current is not more contributing to the V_mem_ setting, locking it at the hyperpolarized state and discontinuing the proper membrane potential rhythmic oscillation.

Conversely, we observed that during the physiological ageing process, as modeled by the Elderly hDF, BK_Ca_ channels expression on plasma membrane and functionality decline. We also observed that the highest percentage of senescent cells (positive to β-gal) has been detected in the Elderly group, while its increase in the diseased subject group (HGPS) is not statistically different in comparison to that of the healthy Young group. Furthermore, as reported in Lattanzi et al. [10], progerin is only detectable in HGPS cells, while lamin A/C levels are not significantly changed in Young and Elderly groups. All these evidences suggest that, despite the cellular proliferation boost is impaired in both normal ageing [10] and in the age-related disease HGPS, the mechanisms that underline this phenotypic expression might be very different.

Besides the fact that cell proliferation is a necessity during development or repairing activity [32] it has already been widely established that its control has also great relevance for neoplastic diseases [30,31,33], age-related pathologies [34] and normal aging process [35]. Preventing the cell to achieve the ideal conditions for replication induces a cellular stress condition that may lead to apoptosis or senescence [34]. Cellular senescence has a crucial tissue-remodeling role in organisms, being recognized and cleared by immune cells. In fact, this is an efficient way to eliminate cells that have accumulated damage during their life, or malignant cells, thus contrasting tumor development [36]. Has been recently proved that oncogenic stress may induce senescence in cells not only increasing the BK_Ca_ channels α subunit expression but also its relocation from the cytoplasm to the membrane, leading to a change on the channel functionality and membrane potential that is recognized and cleared by the immune system cells [35]. Therefore, the overexpression of the BK_Ca_ channels on plasma membrane of HGPS hDF that we observed could be considered as, in addition to a random gene expression dysfunction induced by progerin, an attempt to simulate the senescence of young but diseased cells in order to make them recognize as senescent by the immune system and then eliminate from the organism.

Understanding the role of ion channels expressed in cells affected by HGPS or others age-related diseases may contribute to a better knowledge of the mechanisms underlying these pathological process and, probably give some light to the physiological process of aging as well.

## MATERIALS AND METHODS

### Cell culture isolation and propagation

Cultured human dermal fibroblasts (hDF) were obtained from two juvenile (9 years old) and six young (age range 17-35 years) healthy donors (considered as one group being not statistically different, referred to as “Young group”), four elderly healthy donors (age range 68-86 years, referred to as “Elderly group”), and from two donors carrying a p.G608G *LMNA* mutation (6 and 9 years old, referred to as “HGPS group”).

Primary hDF cultures were from the BioLaM biobank and were established following informed consent and according to local and EU rules. hDF were maintained in Dulbecco’s modified Eagle’s medium (DMEM), supplemented with 20% heat-inactivated fetal calf serum, penicillin (100 units/mL), streptomycin (100 g/mL), 2 mM L-glutamine (all from Sigma Chemical Italy, Milano, Italy) and kept in incubator at 5% CO_2_ and humidified atmosphere at 37°C. Cell cultures were periodically checked for mycoplasma infection.

### Current recordings, data acquisition and analysis

Early passage number (within 5 and 19) hDF at sub-confluence were detached using trypsin-EDTA (0.02%) and suspended in culture medium at room temperature (RT). Within 6 hours cells were transferred to a 35-mm Nunc Petri dish and after 15 min were checked under the microscope to verify their attachment to the dish; culture medium was then substituted with the bath solution. Membrane currents from fibroblasts were measured in the whole-cell configuration of the patch-clamp technique at RT (22–24°C) with a EPC-10 amplifier driven by the Patch Master software (HEKA Instruments, Darmstadt, Germany). Current traces were acquired at digitizing rates of 20 kHz and filtered at 2.9 kHz with an eight-pole low-pass Bessel filter. Voltage steps (20 mV, 100 ms) from -30 to +110 mV were delivered at intervals of 1 s; to inactivate other voltage activated K^+^ currents, the holding potential V_h_ was set to 0 mV. Specific software macros minimized on-line the fast capacitance transients, and performed a tracked leakage subtraction of the current amplitude every 10 s. This software setting allowed the automatic measurement of cell capacitance (C_m_) and resting potential (V_rest_). The patch micropipette tip resistances ranged between 4 and 8 MΩ, when filled with electrode solution. Current amplitude values were considered after reaching the steady-state level, and were averaged to produce a single value for each measurement.

The bath solution (extracellular electrolyte solution) contained (in mM): 133 NaCl, 4 KCl, 2 MgCl_2_, 2 CaCl_2_, 10 4-(2-hydroxyethyl)-1-piperazineethanesulfonic acid (HEPES) and 10 glucose (pH 7.4, NaOH). The electrode solution contained (in mM): 145 KCl, 1 MgCl_2_, 1.8 CaCl_2_, 10 HEPES (pH 7.2, KOH). Solutions containing 10 mM concentration of tetraethylammonium chloride (TEA) were prepared from an adjusted bath solution (123 mM NaCl). Stock solution of Iberiotoxin (Alomone Labs, Jerusalem) in MilliQ water were added to the bath solution at the final saturating concentration of 100 nM. Unless otherwise stated, all chemicals and solutions were purchased from Sigma. Perfusing solutions were delivered at the rate of 1 mL/min trough a metallic cannula connected to a gravity-driven solution exchanger (VM8, ALA Scientific Instruments, Westbury, NY, USA) triggered by a TIB 14 interface (HEKA Instruments, Darmstadt, Germany) under the control of Patch Master 2.15 software.

The data analyses in terms of percentage of cells was estimate by setting as the reference thresholds the mean current amplitude at 110 mV of the Young group minus its STD (i.e. 857.6 – 461.6 = 396; referred to as “Low”); plus the STD (i.e. 857.6 + 461.6 = 1319.2; referred to as “High”) and between these two values (referred to as “Intermediate”). Current densities were obtained by dividing the current amplitude values by cell’s capacitances. Starting from the C_m_ it is straightforward to estimate cellular radius and surface by spherical capacitor-capacitance relation approximating fibroblasts to spherical cells (we recorded all the cells before their complete adhesion to the bottom of the Petri-dish, then their geometry was well approximated by a sphere or an ellipse).

All values were expressed as mean ± SEM. The statistical analysis of the data obtained from patch-clamp experiments was performed by the one-way ANOVA for intra-group homogeneity assessment (not shown). Student’s t test was used to compute the probability values (p) in a two-group comparison. A p threshold of 0.05 was considered for statistical significance.

### Fluorescence assays

Fibroblasts from the Young, Elderly and HGPS groups were seeded at a density of 15.000 cells/well onto imaging 24-well plates with the bottom made from cover glass and suitable for high resolution fluorescence microscopy (Imaging Plate CG 1.5, Miltenyi Biotec GmbH, Germany) and incubated aerobically for 24 hours at 37°C.

Immunofluorescence staining for BK_Ca_ channels expressed on plasma membrane was performed in fixed cells as follow. Fibroblasts adhered in a monolayer culture from Young and HGPS groups were washed with 0.15 M phosphate-buffered saline (PBS) pH 7.0 and fixed with 100% methanol at -20°C for 15 min. Cells were then washed three times in cold PBS and loaded with blocking buffer for 60 min. The BK_Ca_ α subunits were labeled by using the rabbit polyclonal IgG specific to the extracellular amino acids residues KCNMA1-199:213 (Alomone Labs, Jerusalem, Israel) diluted 1:50 in PBS and incubated overnight at 4°C. After washed in PBS, cells were incubated for 1 hour at RT with a donkey anti-rabbit IgG fluorescein 5-isothiocyanate (FITC)-conjugated antibody, diluted 1:400 in PBS. The samples in DAPI-containing mounting medium were observed with the inverse automated optical microscope Eclipse-Ti (Nikon) equipped with Epi-fluorescence filters and driven by the NIS-Elements AR 4.0 software (Nikon). Images were acquired at 200× magnification by using the Retiga-2000RV (Q Imaging, Surrey, BC, Canada) camera.

Quantification of the fluorescence intensity was performed by the NIS-Elements software processing the Regions of Interest (ROI) drawn around ten cells from each visual field acquired from three independent experiments (n=30). To calculate the integrated density, the corrected total cell fluorescence (CTCF) formula has been used: area of selected cell × mean fluorescence of background readings. The mean fluorescence intensity was expressed as arbitrary units (A.U. ± SEM).

Immunofluorescence staining for the BK_Ca_ channels expressed on plasma membrane was also performed in living cells. Living fibroblasts adhered in monolayer culture from Young, Elderly and HGPS groups were double labeled by using the custom synthesized rabbit polyclonal IgG specific to the extracellular amino acids residues KCNMA1-69:86 conjugated with Alexa Fluor 350 (Invitrogen, Life Technologies Europe, Milano, Italy), and the cell-permeant calcium (Ca^2+^) fluorescent probe Fluo-4 AM (Thermo Fisher Scientific Inc., Waltham, MA, USA). Supplemented DMEM solution (500 μl) containing the conjugated primary antibody diluted 1:500 and Fluo-4 AM at concentration 2 μM were added to each well of the imaging 24-well plate. The plates were kept in incubator for 2 hours and then washed three times with DMEM at 37°C. Right after, images were acquired with the appropriate fluorescence filters (DAPI and FITC), and with phase contrast microscopy at 200× magnification.

The percentage of positive hDF to the anti-BK_Ca_ antibody DAPI-conjugated has been calculated dividing the number of cells showing a detectable fluorescence by the total number of cells present in the images acquired in phase contrast from seven wells dedicated to each experimental group in a 24-well plate. The light intensity curve of each image acquired in fluorescence was previously adjusted by the Look-up table (LUTs) tool of the NIS-Elements software as follow: *Gamma parameter* (G) at 20; *input intensity range* in correspondence to the intersection with zero. Quantification of the fluorescence intensity for the Ca^2+^ probe-loaded cells was performed as previously described for the anti-BK_Ca_ antibody FITC-conjugated labeled samples.

Student’s t-test was used to compute the probability values (p) in the two-group comparison and corrected by the Bonferroni test for multiple comparison. A p threshold of 0.05 was considered for statistical significance.

### Cell adhesion and proliferation assay

Early passage number (within 5 and 19) hDF at sub-confluence were detached using trypsin-EDTA (0.02%) and resuspended in supplemented DMEM at RT. Cells were seeded on 24-multiwell plates (CELLSTAR, Greiner bio-one) at density of 0.015×10^6^ with 500 μL of supplemented DMEM. We studied cell adhesion and proliferation in a CO_2_ incubation system integrated within a motorized stage able to perform time-lapse imaging up to several days; the acquisition of images was performed in phase-contrast at 100× of magnification with the inverse automated optical microscope Eclipse-Ti (Nikon).

For adhesion tests, cells were allowed to adhere for 15 min before the observations, which were carried out every 30 min, up to 4 h. The number of adherent and not-adherent hDF cells was determined at 2 hours from plating by classifying them according to morphological parameters such as shape (spherical or non-spherical), structural polarization, the presence of lamellar cytoplasm, leading lamella and focal adhesion. The analysis at 4 hours (data not shown) has been not considered since all the cells has already acquired the morphological requirements to be classified as adherent. The adhesion capability was defined as the number of adherent cells counted in a focal field of 0.68 mm^2^ divided by the total number of cells. A group of cells from each sample population has been treated by IbTx at same concentration used for the current recording experiments (100 nM).

For proliferation tests, cells were allowed to adhere and spread for 96 hours. The day after seeding 100 nM IbTx was diluted in 1 ml of supplemented DMEM per well. Phase-contrast microscopy images were taken at 24, 48, 72 and 96 hours from seeding. The fold-change was determined by counting the number of adherent hDF cells and normalizing each data point to the number of cells at 24 hours. The results of independent experiments were reported as mean ± SEM. Student’s t-test was used to compute the probability values (p) in the two-group comparison and corrected by the Bonferroni test for multiple comparison. A p threshold of 0.05 was considered for statistical significance.

### Senescence-associated β-galactosidase staining

Fibroblasts grown on coverslips were fixed with 2% formaldehyde/0.2% glutaraldehyde for 3 minutes at 22°C and incubated overnight at 37°C in 1 mg/mL 5-bromo-4-chloro-3-indolyl-β-D-galactoside (X-Gal), 40 mM citric acid-sodium phosphate, 5 mM potassium ferricyanide, 5 mM potassium ferricyanide, 150 mM NaCl and 2 mM MgCl_2_. The percentage of hDF with blue staining (positive for SA β-gal activity) has been calculated counting the number of cells from Young, Elderly and HGPS groups present in images acquired in transmission light bright field at 400× magnification from three independent staining.
